# DNA barcoding survey of *Trichoderma* diversity in soil and litter of the Colombian lowland Amazonian rainforest reveals *Trichoderma strigosellum* sp. nov. and other species

**DOI:** 10.1007/s10482-013-9975-4

**Published:** 2013-07-25

**Authors:** Carlos A. López-Quintero, Lea Atanasova, A. Esperanza Franco-Molano, Walter Gams, Monika Komon-Zelazowska, Bart Theelen, Wally H. Müller, Teun Boekhout, Irina Druzhinina

**Affiliations:** 1 CBS Fungal Biodiversity Centre (CBS-KNAW), Utrecht, The Netherlands; 2 TEHO Laboratory, Institute of Biology, University of Antioquia, Medellín, Colombia; 3 Microbiology Group, Research Division Biotechnology and Microbiology, Institute of Chemical Engineering, Vienna University of Technology, Gumpendorferstrasse 1a/E166-5, 1060 Vienna, Austria; 4 Formerly at CBS Fungal Biodiversity Centre (CBS-KNAW), Utrecht, The Netherlands; 5 Department of Biology, Utrecht University, Utrecht, The Netherlands

**Keywords:** Biodiversity, *Hypocrea*, Leaf decomposition, Rhizosphere, Phenotype microarrays, Neotropics

## Abstract

The diversity of *Trichoderma* (Hypocreales, Ascomycota) colonizing leaf litter as well as the rhizosphere of *Garcinia macrophylla* (Clusiaceae) was investigated in primary and secondary rain forests in Colombian Amazonia. DNA barcoding of 107 strains based on the internal transcribed spacers 1 and 2 (ITS1 and 2) of the ribosomal RNA gene cluster and the partial sequence of the translation elongation factor 1 alpha (*tef1*) gene revealed that the diversity of *Trichoderma* was dominated (71 %) by three common cosmopolitan species, namely *Trichoderma harzianum* sensu lato (41 %), *Trichoderma spirale* (17 %) and *Trichoderma koningiopsis* (13 %). Four ITS 1 and 2 phylotypes (13 strains) could not be identified with certainty. Multigene phylogenetic analysis and phenotype profiling of four strains with an ITS1 and 2 phylotype similar to *Trichoderma strigosum* revealed a new sister species of the latter that is described here as *Trichoderma strigosellum* sp. nov. Sequence similarity searches revealed that this species also occurs in soils of Malaysia and Cameroon, suggesting a pantropical distribution.

**Electronic supplementary material:**

The online version of this article (doi:10.1007/s10482-013-9975-4) contains supplementary material, which is available to authorized users.

## Introduction

The Amazon area is one of the largest regions on Earth covered with tropical rain forests and is one of the most biodiverse ecosystems with approximately 60,000 species of vascular plants (Ter Steege et al. 2003; Hoorn et al. 2010). Efforts of multiple research groups have resulted in a considerable increase of our knowledge on the plants occurring in this region (Ter Steege et al. 2003; Pitman et al. 2001; Tuomisto et al. 2003; Baker et al. 2004; Phillips et al. 2004; Kreft et al. 2004; Duque 2004), whereas the diversity and ecology of the microfungi remains relatively underexplored. Fungi play a central role in many ecological processes in forest ecosystems, including decomposition of plant litter and nutrient cycling. Although decomposition rates in tropical forests are typically higher than in temperate forests (Powers et al. 2009) this parameter is highly variable (Hättenschwiler et al. 2011). In tropical as well as cooler regions, colonization by endophytic and epiphytic phyllosphere fungi occur in early stages of decomposition when the loss of litter mass and chemical changes occur most rapidly (Osono and Takeda 2002). Composition and functioning of soil microbial communities are among the key factors that determine decomposition rates (Coûteauxm et al. 1995; Hättenschwiler et al. 2011). Thus the composition of communities of soil micro-organisms present in the nutrient-poor Amazonian rainforests may strongly impact the decomposition process.

*Trichoderma*, a name now generally used in preference over the associated teleomorph *Hypocrea*, is a genus of primarily mycoparasitic/fungicolous filamentous fungi that contains species with great opportunistic potential including the capability to decompose woody and herbaceous materials (see Druzhinina et al. 2011 for references). In-depth molecular evolutionary and taxonomic studies of *Trichoderma* have resulted in the distinction of about 200 currently recognized species (e.g. Samuels Samuels 2006c; Druzhinina et al. 2006, 2010a, 2010b, 2011; Jaklitsch 2009, 2011; Atanasova et al. 2013a). Species recognition in *Trichoderma* is usually based on the application of the genealogical concordance phylogenetic species recognition concept (Taylor et al. 2000) based on the partial sequences of the translation elongation factor 1-alfa (*tef1*), endochitinase *chi18*-*5*, calmodulin (*cal1*) and other loci. The concept allows an assignment of the species rank to the clade that is apparent on at least two single-loci phylograms and is not contradicted by the others. The relatively well detailed molecular phylogeny of *Trichoderma* resulted in the development of reliable tools for infrageneric DNA barcoding and for the recognition of new species (Druzhinina et al. 2005; Kopchinskiy et al. 2005; Atanasova et al. 2013a). *Trichoderma* diversity has been previously explored in Colombia, but only three species have hitherto been reported from the Colombian Amazon region, namely *T. virens, T. asperellum* and *T. harzianum* (Hoyos-Carvajal et al. 2009a).

The development of microfungal communities in litter bags was studied in primary and secondary lowland rainforests in two regions of Colombian Amazonia, viz. Araracuara and Amacayacu, which are approximately 600 km apart, using a culturing approach to reveal the fungal succession of leaf litter in forests at different stages of regeneration. The fungi were isolated from the litter bags after different periods of decomposition. In the Amacayacu region this litter-related diversity was compared to that present on rootlets of *Garcinia macrophylla* (*Clusiaceae*), a tree species that occurred in all four Amacayacu plots. The objective of the study we present here was to investigate the diversity of *Trichoderma* and discuss the potential of these fungi for the decomposition of leaf litter in lowland tropical rainforest.

## Materials and methods

### Study area

The studied forests in Colombian Amazonia belong to the tropical humid forest according to the life zone definition of Holdridge (Holdridge et al. 1971; Holdridge 1982) having an equatorial superhumid climate without a dry season (Type Afi of Köppen, 1936, cited by Duivenvoorden and Lips 1993). The average annual temperature is approximately 25 °C with over 100 mm precipitation every month resulting in an average annual rainfall of 3,100–3,300 mm (Tobón Marín 1999). Two locations were selected in the Middle Caquetá region. The first location was on the lower terrace of the Caquetá River, near Araracuara community (0°37′ S, 72°23′ W). The seven plots studied at this location are part of a mosaic of primary and secondary forests and of agricultural fields originating from slash-and-burn agriculture (chagras) at different stages of regeneration (López-Quintero et al. 2012). A second location in this region comprised a mature forest characterized by the presence of a dipterocarp tree species, *Pseudomonotes tropenbosii* (Dipterocarpaceae, Londoño et al. 1995), located about 50 km downstream from the Araracuara region in Peña Roja (00°34′ S, 79°08′ W) at 200–300 m altitude (López-Quintero et al. 2012). The second location was chosen in the National Park Amacayacu (3°25′ S, 70°08′ W), which covers 293,500 ha of tropical humid forest. Here, two terra firme (i.e. non-flooded) plots and two várzea (i.e. flooded) plots were selected, each containing a mature and a regenerating forest. Full details on the forests studied and the plots selected are provided by López-Quintero et al. (2012).

### Litter decomposition experiments and isolation procedure

Fresh mixed leaf litter from dominant trees occurring in the plots was collected from the forest floor. The litter was air-dried, weighed, packed in 27 litterbags with a mesh size of 1 mm^2^ at each location, thus in total 108 litterbags for all four locations, and placed directly on top of the litter layer at the forest floor of the respective plots. One litter bag was recollected after four different times of exposure, namely after 4–6, 9, and approximately 12 and 17 months of exposure on the forest floor. Microfungi were isolated from particles of fresh and decomposed leaf litter samples using a soil-washing method modified after Gams and Domsch (1967). Briefly, three grams of fine litter fragments were taken from the litter bags, and washed three times for 5 min each time with 500 ml sterile distilled water using strong mechanical agitation. The washed particles were blotted dry aseptically and four of them with an approximate size of 4 mm^2^ were placed in each of ten Petri dishes containing 2 % water agar. Thus 40 litter particles were used in total for each plot and time of isolation. After incubation for 7 days at 25 °C in the dark, mycelia growing out from the litter particles were picked and transferred to cornmeal agar (CMA, Difco) and further purified. In addition, 10 rootlets of *Garcinia macrophylla* were sampled from each plot at the Amacayacu location for the isolation of microfungi using the same isolation method, but plating approximately 10-mm-long and 1-mm-diam root fragments. All microfungi were preliminarily identified morphologically and subsequently by ITS-based DNA barcoding using sequence similarity search as available at NCBI portal (http://www.ncbi.nlm.nih.gov). Here we present the observed diversity of *Trichoderma* isolates (Table 1), whereas a full study on all fungal isolates will be presented elsewhere.

**Table 1:** Isolates of *Trichoderma* used in this study. GenBank accession numbers of ITS1 and 2 (rRNA) are included

	Code	CBS code	Forest type	Roots/leaves	ITS1 and 2 GenBank accession nr.
Amacayacu					
*Trichoderma epimyces*	MF50		Mature	Roots	JX416579
*T. asperellum*	MF7c		Mature	Roots	JX416485
	MFIS13a		Mature	Roots	JX416495
	MF13c		Mature	Leaves	JX416534
*T. asperelloides*	MFIS16a		Flooding	Leaves	JX416498
	MFIS16b		Flooding	Leaves	JX416499
	MF10a		Mature	Leaves	JX416492
*T. hamatum*	FPF2		Flooding	Leaves	JX416540
	MFIS59		Flooding	Leaves	JX416509
*T. harzianum* s.s.	MF2b		Mature	Roots	JX416567
	MF3c		Mature	Roots	JX416572
	FPF38a		Flooding	Roots	JX416577
	FPF6b		Flooding	Roots	JX416482
	MFIS4e		Flooding	Roots	JX416478
	RF6a		Successional	Leaves	JX416481
	FPFh8		Flooding	Roots	JX416543
	MFIS6d		Flooding	Roots	JX416484
	MF3a		Mature	Roots	JX416570
	MFIS4c		Flooding	Roots	JX416575
	RF2a		Successional	Roots	JX416566
	MFIS4a		Flooding	Leaves	JX416573
*T.* cf. *harzianum*	MFIS4b		Flooding	Leaves	JX416574
	FPFh9		Flooding	Roots	JX416537
	FPF13b		Flooding	Leaves	JX416496
	RF7b		Successional	Leaves	JX416486
	RF10b		Successional	Leaves	JX416493
	RF1d		Successional	Leaves	JX416564
	MF2c		Mature	Roots	JX416568
	MF3b		Mature	Roots	JX416571
	MF7a		Mature	Roots	JX416485
	RF27		Successional	Roots	JX416501
	FPF38b		Flooding	Roots	JX416578
*T. inhamatum*	MF51b		Mature	Leaves	JX416507
*T. koningiopsis*	RF60		Successional	Leaves	JX416510
	RF16c		Successional	Leaves	JX416500
	FPF2d		Flooding	Leaves	JX416569
	FPF12b		Mature	Leaves	JX416494
	FPF12a		Flooding	Leaves	JX416576
	MF2		Mature	Roots	JX416544
	RF4d		Successional	Roots	JX416580
	FPF6c		Flooding	Roots	JX416483
*T. spirale*	FPF5c		Flooding	Roots	JX416479
	MFIS5b		Flooding	Roots	JX416581
	FPF7		Flooding	Roots	JX416539
	MF14b		Mature	Roots	JX416497
	FPFh5		Flooding	Roots	JX416535
	RF9a		Successional	Roots	JX416491
	MF8c		Mature	Leaves	JX416490
	FPF8b		Flooding	Leaves	JX416489
	RF5d		Successional	Leaves	JX416480
	MFIS52		Flooding	Leaves	JX416508
	FPF8a		Flooding	Leaves	JX416488
	MF33		Mature	Leaves	JX416545
	FPF17b		Flooding	Leaves	KC176364
	RF57		Successional	Roots	KC176362
*T. virens*	RF1c		Successional	Leaves	JX416563
	RF1e		Successional	Leaves	JX416565
	RF32		Successional	Leaves	KC176361
	MFIS29a		Flooding	Leaves	JX416503
	MFIS29b		Flooding	Leaves	JX416504
	FPF1b		Flooding	Leaves	KC176360
*T.* sp. DAOM 229888	FPFh10		Flooding	Leaves	JX416538
*T.* sp. DAOM 229990	FPFh19		Flooding	Leaves	JX416541
	FH6-16		Flooding	Leaves	n/a
Araracuara					
*T.* cf. *harzianum*	P1-9(3)		Successional	Leaves	KC176363
	P3-5(40)		Successional	Leaves	JX416552
	P6-9(69)		Mature	Leaves	JX416559
	P4(107)		Successional	Leaves	JX416517
	P4(108)		Successional	Leaves	JX416546
	P6(92)		Mature	Leaves	JX416514
	P6(81)		Mature	Leaves	JX416513
	P6(69a)		Mature	Leaves	JX416512
	PR(28)		Mature	Leaves	JX416502
	P6(98)		Mature	Leaves	JX416516
	P6(67)		Mature	Leaves	JX416511
	P6(97)		Mature	Leaves	JX416515
	P4(119)		Successional	Leaves	JX416519
	P4(117)		Successional	Leaves	JX416518
	P6-8(71)		Mature	Leaves	JX416558
	P6(100a)	CBS102788	Mature	Leaves	JX416520
*T. koningiopsis*	P3-2(45)		Successional	Leaves	JX416549
	P3-2(46)		Successional	Leaves	JX416550
	P3-7(93)		Successional	Leaves	JX416553
	P6-2(82)		Mature	Leaves	JX416556
*T. spirale*	5a		Mature	Leaves	JX416582
	P6-5(77)		Mature	Leaves	JX416557
	P3(155)	CBS102811	Successional	Leaves	JX416525
	P6(53)	CBS102814	Mature	Leaves	JX416527
*T. strigosellum* sp. nov.	P4(166)		Successional	Leaves	JX416554
	P1-2(25)		Successional	Leaves	JX416547
*T. strigosum*	P3-3(43)	CBS102804	Successional	Leaves	JX416551
*T.* cf. *rogersonii*	P4-4(64)		Successional	Leaves	JX416555
	P1-6(13)		Successional	Leaves	JX416548
Peña Roja					
*T.* cf. *harzianum*	PR-10(85)		Mature	Leaves	JX416561
	PR(33)		Mature	Leaves	JX416505
	PR(38)		Mature	Leaves	JX416506
*T. koningiopsis*	PR-4(97)		Mature	Leaves	JX416560
*T. strigosellum* sp. nov.	PR(220)	CBS102816	Mature	Leaves	JX416528
	PR(216)	CBS102818	Mature	Leaves	JX416530
	PR(213)	CBS102805	Mature	Leaves	JX416522
	PR(12)	CBS102806	Mature	Leaves	JX416523
	PR(211)	CBS102817	Mature	Leaves	JX416529
*T. strigosum*	PR(1)	CBS102807	Mature	Leaves	JX416524
	PR(2)	CBS102812	Mature	Leaves	JX416526
*T.* cf. *stilbohypoxyli*	PR(218)	CBS102820	Mature	Leaves	JX416532

### DNA extraction, PCR amplification and sequencing

Genomic DNA of *Trichoderma* was isolated using the QIAGEN DNeasy^®^ Plant Mini Kit following the manufacturer’s protocol. The ITS1, 5.8S rRNA and ITS2 regions of the ribosomal RNA (rRNA) gene cluster were amplified using the primers ITS1 and ITS4 (White et al. 1990), sequenced using an ABI 3700 capillary sequencer (PE Biosystems) and further analyzed using the Lasergene software package (DNASTAR Inc.). Fragments of *chi18*-*5* (GH18 chitinase CHI18-5, previously called *ech42*), *cal1* (calmodulin) and *tef1* (translation elongation factor 1 alpha) were amplified as described previously (Druzhinina et al. 2008; Jaklitsch et al. 2006). *Chi18*-*5* is a protein coding fragment, *cal1* has one intron and the *tef1* fragment contains two introns, one complete and one partial exons. PCR fragments of these genes were purified (PCR purification kit, Qiagen, Hilden, Germany) and sequenced at Eurofins MWG Operon (Ebersberg, Germany).

### DNA barcoding

All sequences were aligned for each locus separately and grouped to phylotypes using MEGA 5 software. Unique phylotypes were identified as follows: ITS1 and 2 sequences were identified using the oligonucleotide barcode program *TrichOK*EY (www.isth.info; Druzhinina et al. 2005). Ambiguous cases were then subjected to the sequence similarity search tool blastn against the NCBI GenBank database (http://www.ncbi.nlm.nih.gov). All isolates that were not resolved by ITS1 and 2 sequences (*T. longibrachiatum* and *H. orientalis*, section *Trichoderma* and others) were then identified by the analysis of the fourth intron of *tef1* using a sequence similarity search against the NCBI GenBank and *Tricho*BLAST (www.isth.info, Kopchinskiy et al. 2005) databases. The NCBI accession numbers for ITS sequences obtained in this study are listed in Table 1.

### Phylogenetic analyses

DNA sequences were aligned with CLUSTAL X version 2.1 (Thompson et al. 1997; Larkin et al. 2007) and visually verified with GeneDoc version 2.6 (Nicholas and Nicholas Nicholas and Nicholas HB Jr 1997). Ambiguous fragments of the alignment were removed with the gBlocks server for the selection of less stringent options (Talavera and Castresana 2007). The loci used in this study were previously checked for absence of intragenic recombination (Druzhinina et al. 2008). Neutral evolution was tested by linkage disequilibrium-based statistics and Tajima’s test as implemented in DnaSP 4.50.3 (Rozas et al. 2003). The interleaved NEXUS file was formatted using PAUP*4.0b10 (Swofford 2002). The best nucleotide substitution model for each locus was determined using jMODELTEST (Posada 2003) and the unconstrained GTR + I + G nucleotide substitution model was applied to all loci. Metropolis-coupled Markov chain Monte Carlo (MCMC) sampling was performed using MrBayes v. 3.0B4 (Ronquist and Huelsenbeck 2003) with two simultaneous runs of four incrementally heated chains that performed for 1–3 millions of generations. The number of generations for each dataset was determined using the AWTY graphical system (Nylander et al. 2008) to check the convergence of MCMC. Bayesian posterior probabilities (PP) were obtained from the 50 % majority rule consensus of trees sampled every 100 generations after removing the first trees (300–500 depending on the locus). PP values lower than 0.95 were not considered significant (Leaché and Reeder 2002).

### Morphological examination

Growth rates of the isolates were assessed after inoculation near the margin of 9-mm-diameter Petri dishes using three different media, viz. CMA (Difco cornmeal agar supplemented with 2 % D(+)-glucose-monohydrate. i.e. CMD), SNA (synthetic nutrient-poor agar), and OA (oatmeal agar; for recipes of the latter two media see Gams et al. 2007) and incubated in the dark at 24, 27, 30, 33, and 36 °C. The colony radius was measured daily until the colonies reached the opposite side of the Petri dish. Colony color was characterized according to the Methuen Handbook of Color (Kornerup and Wanscher 1983).

Conidial dimensions, based on 25 measurements for each isolate-medium combination, were made using photographs made with a Zeiss Axioskop 2 and interference contrast using a 63 ×/1.5 Plan-Neofluar objective and equipped with a Nikon Ds-Fi1 camera. Images were processed by the Nikon NIS-elements D software package. Conidiophore structures and measurements of phialides and hyphal cells were recorded at 2,000× magnification with a Wild camera lucida. Colony features were studied with a Leica NZ FLIII binocular microscope.

For scanning electron microscopy parts of the colonies growing on OA agar plates were fixed in 3 % glutaraldehyde/PBS and postfixed in 1 % osmium tetroxide. After dehydration through an ethanol and acetone series, the fungal cells were critical point-dried followed by Pt/Pd sputter coating. Cells were viewed with a field emission scanning electron microscope at 5 kV (FEI, Eindhoven, The Netherlands) as described by Teertstra et al. (2009).

### Phenotype microarrays

Growth of putative new species and respective reference strains (*T. strigosum,* *T. strigosellum* sp. nov. and *T*. sp. C.P.K. 3606) was analyzed on 95 carbon sources using the Biolog Phenotype MicroArray system for filamentous fungi (Biolog Inc.) as described before (Druzhinina et al. 2006; Friedl et al. 2008; Atanasova et al. 2010). Incubation was performed at 12 h cyclic illumination as 25 °C. Statistical analyses were performed using Statistica 6.1 (StatSoft. Inc.).

## Results

### *DNA barcoding* of Trichoderma *diversity revealed twelve known and four putatively new taxa*

Ninety-four (88 %) out of 107 strains were recognized as 10 species of *Trichoderma* by using the oligonucleotide barcode programs *TrichO*Key (Druzhinina et al. 2005) and *Tricho*BLAST based on ITS1 and 2 and *tef1* phylotypes, respectively, (Table 2). The remaining 13 strains could not be reliably identified. Two isolates (FPFh19 and FH6-16) and one isolate (FPFh10), all from rootlets of *Garcinia macrophylla* in the Amacayacu flood plain forests (i.e. várzea), had identical *tef1* phylotypes to DAOM 229990 (NCBI GenBank EU280015) and DAOM 229888 (EU280054), respectively, that were detected previously by Hoyos-Carvajal et al. (2009a) in rain forest soils of Peru. DAOM 229990 represents a putative new species in section *Trichoderma*, while DAOM 229888 formed a long isolated lineage distantly related to *T. helicum* (Hoyos-Carvajal et al. 2009a). Both species had previously been found in forest soil in Loreta near Iquitos, Perú (Hoyos-Carvajal et al. 2009a). Three other isolates [i.e. P4(129b), P4-4(64) and P1-6(13)] obtained from a 30-year-old secondary forest plot and a recently cut down primary forest plot in the Araracuara region have an ITS1 and 2 + *tef1* haplotype related to *T. rogersonii* in the ‘Small Koningii Branch’ (Samuels et al. 2006b) and thus were assigned as *T.* cf. *rogersonii*. Seven isolates CBS 102805, 102806, 102816, 102817 and 102818 from leaf litter decomposing for six months in the mature *Pseudomonotes tropenbosii* (*Dipterocarpaceae*) forest in Peña Roja, as well as strains P1-2(25) and P4(166) isolated from 17-month-old litter in a recently cut down forest (P1) and a 30-year-old secondary forest plot (P4) in Araracuara shared highly similar ITS1 and 2 phylotypes and were attributed to section *Trichoderma* by *TrichO*Key, but no species identification was obtained. Sequence similarity based on 291 nt of the *tef1* large intron determined that these isolates are most closely related to *T. strigosum* (89–90 % of similarity) while other species of section *Trichoderma* showed only 85 % similarity or less. Therefore they were tentatively identified as *T*. cf. *strigosum* (see below). Interestingly, three true *T. strigosum* isolates have also been detected by *TrichO*Key and confirmed by *tef1* (Tables 1, 2).

**Table 2:** Distribution of *Trichoderma* species in Colombian Amazonia according to region, forest type, substrate and decomposition length

Species	Region				Forest type			Substrate		Decomposition (months)					
	All	Amacayacu	Araracuara	Peña Roja	Flooded	Mature	Successional	Litter	Roots	0	4	6	9	12	17
*T. asperellum*	3	3			1	2		2	1	2			1		
*T. asperelloides*	3	3			2	1		3		1	1		1		
*T.* sp. DAOM 229990	2	2			2			2			2				
*T*. sp. DAOM 229888	1	1			1			1		1	1				
*T. epimyces*	1	1				1			1						
*T. harzianum* s.s.	2	2				2			2	2					
*T. hamatum*	2	2			2			2		1	1				
*T.* cf. *harzianum*	41	23	16	2	11	17	13	28	13	18	4	13			6
*T. inhamatum*	1	1				1	0	1			1				
*T. koningiopsis*	14	9	4	1	3	4	7	11	3	5	3		1		5
*T.* cf. *rogersonii*	3		3				3	3				1			2
*T. spirale*	18	15	3		7	6	5	11	7	8	4	2	2	1	1
*T. strigosum*	3		1	2		2	1	3				2			1
*T. virens*	6	6			3		3	6		4	2				
*T. strigosellum* sp. nov.	7		2	5		5	2	7				5			2
Total	107	68	29	10	32	41	34	80	27	42	19	23	5	1	17

### *A few cosmopolitan species dominate the* Trichoderma *mycoflora in the Amazonian leaf litter*

Three species comprising 68 % of the isolates dominated the diversity of *Trichoderma* in the Amazon forests investigated, namely the *T. harzianum* complex (38 %), *T. spirale* (17 %) and *T. koningiopsis* (13 %) (Table 2). Note that these species were dominantly isolated from either leaf litter or *Garcinia* rootlets, and from terra firme, várzea and successional forests (Table 2). Interestingly, the *T. harzianum* species complex was represented by at least three genetically distinct phylogenetic species, namely *T. inhamatum* (1 strain), *T. harzianum* sensu stricto (2 strains) and *T.* cf. *harzianum* (=*H*. ‘pseudoharzianum’ sensu Druzhinina et al. 2010a, 41 strains). The next frequent species are: the putatively novel taxon related to *T.* *strigosum* (=*T. strigosellum* sp. nov., see below) (7 strains); *T. virens* (6 strains); *T. asperellum* (3 strains) and *T. asperelloides* (3 strains). All other taxa were detected not more than twice. Thirteen *Trichoderma* species were isolated from litter bags at different stages of decomposition (Table 2), while only six taxa were detected from rootlets of *Garcinia macrophylla* (Table 2). The *Trichoderma* community from the decomposing litter was less diverse than from fresh to little-decomposed leaves (Table 2). Fresh leaf litter and relatively little-decomposed leaves of 4–6 months yielded 84 isolates, compared to six isolates from 9 to 12 months-old leaves and 17 isolates from 17-months-old leaves. *T. asperellum*, *T. asperelloides*, *T*. sp. DAOM 229888, *T. harzianum* sensu stricto and *T. hamatum* were also isolated at least once from fresh leaf litter.

All dominantly found species occurred in both Amacayacu and Araracuara regions. Twelve species were observed in the Amacayacu plots and only six in Araracuara including Peña Roja. *T. strigosum* and *T. strigosellum* sp. nov. (see below) were detected in Araracuara and Peña Roja, and *T*. cf. *rogersonii* was only observed in Araracuara. *Trichoderma epimyces, T. virens, T. asperelloides, T. asperellum, T. hamatum, T. inhamatum*, *T.* sp. DAOM 229888 and *T.* sp. DAOM 229990 were isolated in Amacayacu and not in Araracuara. No clear distinction was apparent between the number of isolates obtained from primary and secondary forests, nor between those isolated from terra firme and várzea forests in Amacayacu (Table 2).

### *Genealogical concordance phylogenetic species recognition confirms* T. strigosellum *sp. nov.*

**Fig. 1:**
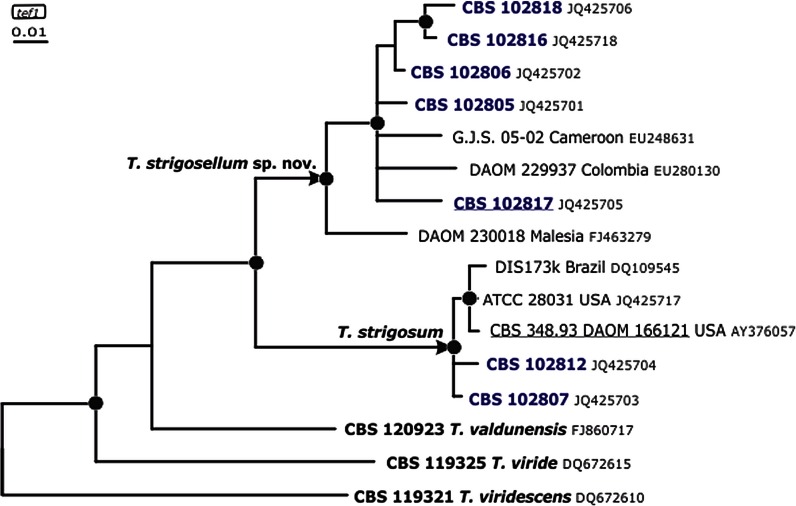
Position of *T. strigosellum* sp. nov. in the *tef1* Bayesian phylogenetic tree. *Black circles* indicate nodes supported by posterior probabilities higher than 0.94. *Arrows* indicate branches that lead to species clades. Numbers correspond to GenBank accessions

**Fig. 2:**
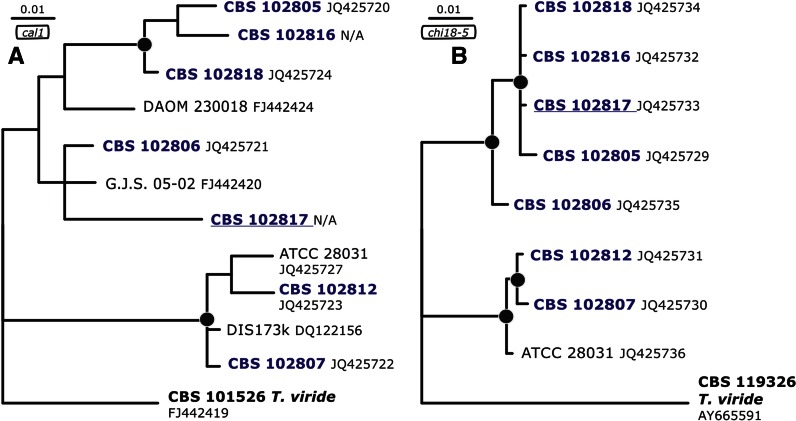
Position of *T. strigosellum* sp. nov. in the *cal1* (**a**) and *chi18–5* (**b**) Bayesian phylogenetic trees. *Black circles* indicate nodes supported by posterior probabilities higher than 0.94. Numbers correspond to GenBank accessions. N/A- accession not available

To reveal the exact phylogenetic position of isolates identified as *T.* cf. *strigosum* in section *Trichoderma* we applied the exact sequence of the 4th large intron of *tef1* (as retrieved by *Tricho*MARK, www.isth.info, Druzhinina et al. 2005) to sequence similarity search (blastn) against NCBI GeneBank. The application of the precise intron sequence without flanking coding areas is necessary to get the most accurate identification result that is not biased by strong similarities of less polymorphic coding regions (exons). The taxonomy report obtained from this search revealed that besides *T. strigosum*, the query isolates are related to *Hypocrea valdunensis* (1 hit), *T. viride* (teleomorph *H. rufa*, 79 hits) and *T. viridescens* (4 hits) (listed in decreasing similarity). The Bayesian phylogram constructed with *tef1* sequences of *T. strigosum* and the query isolates (Fig. 1) demonstrated that *T. strigosum* and *T*. cf. *strigosum* are monophyletic and both belong to a statistically supported clade together with *T. valdunensis* and *T. viride*, while *T. viridescens* is the most distanced genetic neighbor of them. Interestingly, isolates of *T. strigosum* and *T.* cf. *strigosum* formed two statistically supported subclades what allowed us to hypothesize that they may represent two sister species. To test this we constructed Bayesian phylograms based on *cal1* and *chi18*-*5* phylogenetic markers (both unlinked to *tef1*, see *Trichoderma* genomes on Mycocosm portal of DOE JGI and Kubicek et al. 2011). This analysis demonstrated that both subclades were also present on *chi18*-*5* and *cal1* phylogenetic trees. The same result, the two statistically supported subclades corresponding to *T strigosum* and *T. strigosellum* sp. nov. were also present of a concatenated phylogram with *tef1*, ITS1 and 2, *cal1* and *chi18*-*5* loci (Supplementary materials). Thus, the isolates of *T*. cf. *strigosum* fulfill the criteria of the genealogical concordance phylogenetic species recognition concept (Taylor et al. 2000) and represent a new species described below as *T. strigosellum* sp. nov. The phylogenetic position of isolates identified as *T. strigosum* was also confirmed by this analysis (Figs. 1 and 2).

A sequence similarity search conducted for all sequences of this new species revealed further strains of the species that until now had been identified as *T. strigosum.* These had been isolated from Malaysia (DAOM 230018), Colombia (DAOM 229937) and Cameroon (G.J.S. 05-02) suggesting that this new species has a broad, probably pantropical distribution (Figs. 1, 2). Its sibling species, *T. strigosum*, found during our studies in the same regions and habitats in Colombia as the new taxon, is further known from Brazil, forest soil from North Carolina, USA, soil under *Theobroma cacao* trees in Pastaza district, Peru, and from a forest in Turkey (Ismail Erper, Lea Atanasova, Irina Druzhinina, unpublished data), thus suggesting a geographically broad distribution for this species.

### *Physiological profiling of* T. strigosellum *sp. nov. and* T. strigosum

**Fig. 3:**
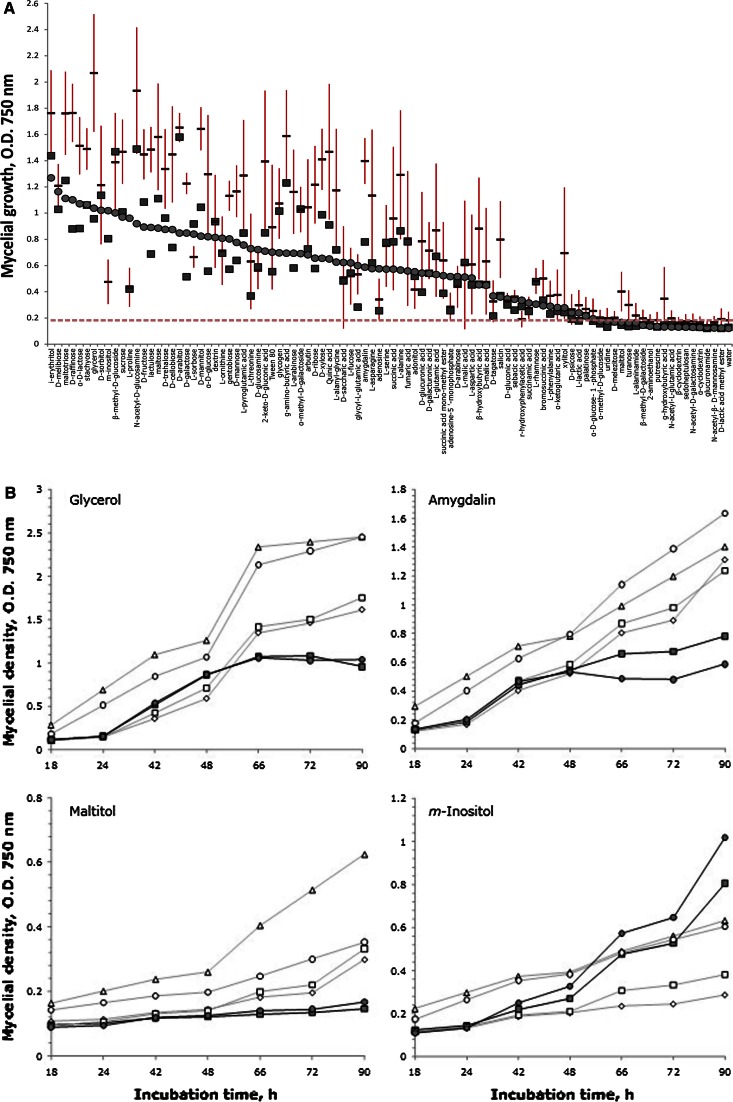
Metabolic profiling of *Trichoderma strigosum* sp. nov. and *T. strigosellum.* A. Biolog phenotype growth profiles of *T. strigosum* and *T. strigosellum* sp. nov. on 95 carbon sources after 90 h of incubation (25 °C) of strains isolated from leaf litter in Colombian Amazon forests: *Red stripes* represents the mean value for four strains of *T. strigosellum* sp. nov. (CBS 102805, CBS 102816, CBS 102806 and CBS 102817). *Filled squares* and *circles* correspond to *T. strigosum* CBS 102812 and CBS 348.93. A dashed line indicates the water control. Standard deviations are given by *vertical bars*. B. Growth rates of *T. strigosum* and *T. strigosellum* sp. nov. on selected carbon sources. *Dark* and *gray* lines represent strains of *T. strigosum* and *T. strigosellum* sp. nov., respectively: *T. strigosellum* sp. nov. CBS 102805 (*rhomb*), CBS 102816 (*square*), CBS 102806 (*triangle*), CBS 102817 (*circle*), and *T. strigosum* CBS 102812 (*square*) and CBS 348.93 (*circle*), respectively

**Fig. 4:**
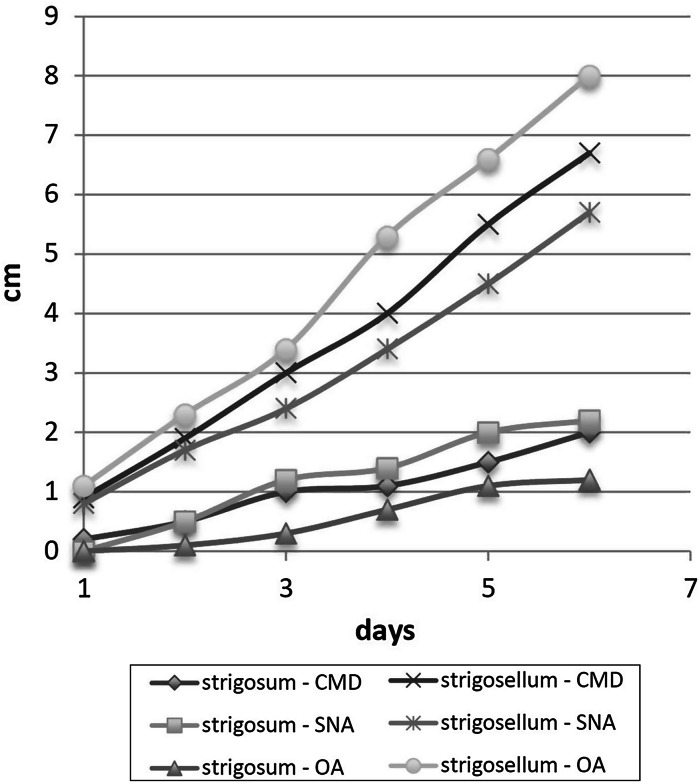
Growth rates of *Trichoderma strigosum* CBS 102807 and *T.strigosellum *sp. nov. CBS 102817 at 33 °C

We applied BIOLOG Phenotype MicroArrays with FF Phenotype microplates to further test whether *T. strigosellum* sp. nov. and *T. strigosum* are physiologically similar or may be distinguished by phenotypic characters. Carbon utilization by *T. strigosellum* sp. nov. was rather similar to *T. strigosum* as both could grow on almost all tested carbon sources (Fig. 3a). *M*-inositol, however, is hardly utilized by *T. strigosellum* sp. nov. In most cases *T. strigosellum* sp. nov. showed better growth than *T. strigosum*, especially on the best utilized carbon sources, such as d-lactose, *N*-acetyl-d-glucosamine, d-maltotriose, d-raffinose, maltose, lactulose, and stachyose. For some compounds, such as d-melibiose, d-sorbitol, l-ornithine, l-threonine, l-fucose, d-saccharic acid, glycyl-l-glutamic acid, and adonitol, growth was variable (Fig. 3a), but rather strain- and not species-dependent. Thus, the largest differences in hyphal growth were observed on carbon sources such as glycerol, amygdalin, *m*-inositol and maltitol (Fig. 3b). This analysis further supported our above conclusion on divergence between *T. strigosellum* and *T. strigosum*. Furthermore, and in line with it, linear growth rates at 30 and 33 °C were higher for *T. strigosellum* sp. nov. compared to *T. strigosum* (Fig. 4).

### *Development of an ITS1 and 2 oligonucleotide barcode for* T. strigosellum *sp. nov.*

**Fig. 5:**
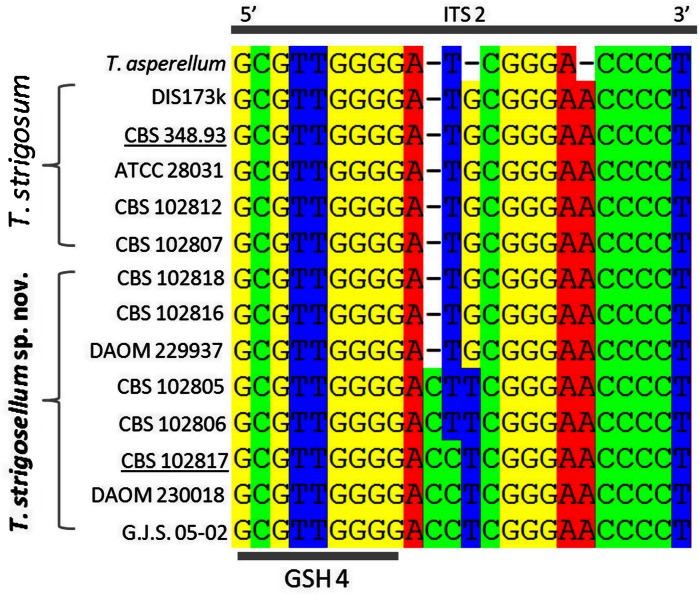
ITS2 based oligonucleotide barcodes for *T. strigosum* and *T. strigosellum* sp. nov. GSH4 corresponds to the genus-specific hallmark as indicated in Druzhinina et al. 2005

We compared ITS1 and 2 phylotypes of *T. strigosellum* sp. nov. and *T. strigosum* and found that five out of eight available sequences for *T. strigosellum* sp. nov. had a ‘species-specific’ oligonucleotide barcode in the 5′ area of the ITS2 locus that immediately follows the genus-specific hallmark four (Druzhinina et al. 2005). Compared to *T. strigosum* this hallmark contained one indel (an extra C), one T → C transition and one G → T transversion (Fig. 5). However, three strains of *T. strigosellum* sp. nov. displayed a phylotype identical to that of *T. strigosum* (Fig. 5). Thus the ITS barcode alone cannot reliably identify both species, but may attribute them to the *T. strigosum* clade. The two species are reliably differentiated in phylogenetic analyses of the *tef1* large intron.

## Discussion

Fungi play diverse roles in the functioning of tropical forest ecosystems. Unfortunately, biodiversity studies on microfungi in Colombian Amazonian rainforests are still sparse. Here we investigated the diversity of *Trichoderma* species in decomposing leaf litter in a series of primary and secondary Amazon forests from the Araracuara and Amacayacu regions that were recently studied for mushroom and plant diversity (López-Quintero et al. 2012). The macrofungal diversity differed considerably between these two Amazon regions, but also between primary and secondary forests, as well as between flooded and non-flooded forests (López-Quintero et al. 2012).

Hitherto, only three *Trichoderma* species have been reported from Colombian Amazonia, namely *T. virens, T. asperellum* and *T. harzianum* (Hoyos-Carvajal et al. 2009a). Thus the 15 *Trichoderma* species that we report from Colombian Amazonia, including four putative new species, show that the microfungal diversity of these forests deserves further exploration. Other species, such as *T. atroviride, T. brevicompactum, T. erinaceus, T. hamatum, T. inhamatum, T. koningii, T. koningiopsis, T. longibrachiatum, T. reesei, T. viridescens,* together with a few so-far undescribed species have been reported from other parts of the country (Veerkamp and Gams 1983; Hermosa et al. 2000, 2004; Kraus et al. 2004; Ortiz and Orduz 2000; Lee and Hseu 2002; De Souza et al. 2006; Samuels et al. 2006b; Mendez and Viteri Méndez and Viteri 2007; Hoyos-Carvajal et al. 2009a) with *T. harzianum*, *T. asperellum* and *T. asperelloides* (reported as *T. asperellum* ‘B’) being most commonly isolated, followed by *T. brevicompactum* (Hoyos-Carvajal et al. 2009a, 2009b).

*Trichoderma* cf. *harzianum*, *T. koningiopsis*, and *T. spirale* occurred in fresh leaves, but also in young to 17-months-decomposing leaves. The repeated presence of *T.* cf. *harzianum, T. spirale,* *T. koningiopsis* and *T. virens* (isolated more than three times) in freshly collected leaf litter suggests that these species may occur as leaf endophytes. Endophytic colonization of epigeous parts of tropical plants is known for several apparently rare *Trichoderma* species (see Druzhinina et al. 2011 for references) that we did not detect in this study. There are also indications that numerous common environmentally opportunistic species, such as *T.* cf. *harzianum* and *T. hamatum* may also become endophytes (Chaverri et al. 2011, Chaverri and Samuels, 2013) and *T. harzianum* s.s. and *T. asperellum* were reported as endophytic in bean stem tissue by Hoyos-Carvajal et al. (2009b). However, our understanding of the functional diversity of *Trichoderma* species in the Colombian tropical lowland Amazon remains limited, especially with respect to this switch between endophytic and saprotrophic life styles. *Trichoderma* can be mycotrophic feeding on living and dead fungal biomass. Recent genomic and transcriptomic studies (Kubicek et al. 2011, Druzhinina et al. 2011, Atanasova et al. 2013b) have proven that mycotrophy is the major genetic basis that allows *Trichoderma* to establish itself in a diversity of habitats ranging from biotrophy on plants and animals to exclusive saprotrophy. According to the concept of Kubicek et al. ( 2011) and Druzhinina et al. (2011), *Trichoderma* is initially fungicolous and this lifestyle gave rise to a number of derived nutritional strategies including biotrophy and saprotrophy. In this study we demonstrated that *Trichoderma* is present in the community of leaf litter-decomposing fungi in Colombian Amazonia. However, whether *Trichoderma* is a primary decomposer in this ecosystem or whether it follows other fungi remains unresolved. A pioneering occurrence of many *Trichoderma* species has been repeatedly observed in soils of unstable ecosystems (summarized by Domsch et al. 2007). Moreover, *Trichoderma* species together with fungi such as *Mucor hiemalis* and *Absidia glauca*, were found to appear later in the fungal succession of decomposing *Swida* leaves (Osono 2005). These findings demonstrate that *Trichoderma* spp. may play their role during various stages of litter decomposition.

It appears remarkable that the diversity of *Trichoderma* in the biodiversity-rich ecosystem of the tropical lowland Amazon forest was found to be dominated by a group of cosmopolitan species with high opportunistic potential, such as *T*. cf. *harzianum*, *T*. *spirale* and *T. koningiopsis* (Atanasova et al. 2013a). Similar observations were made by Migheli et al. (2009) on Sardinia located in the Mediterranean hotspot of biodiversity where *Trichoderma* diversity did not contain any endemic species and was dominated by the same species as detected in the current study. Migheli et al. (2009) speculated on the relative role of human activity that favors establishment of invasive *Trichoderma* species and harms the presumed endemic community of the otherwise unique and species rich environment. The results of the current study further support the view that a number of *Trichoderma* species that are most frequently detected in soil and litter form invasive communities that establish in various ecosystems. However, the interaction between the later ‘strong’ *Trichoderma* species and local infrageneric communities requires further investigation.

The likely pantropical *T. strigosellum* sp. nov. differed ecophysiologically from its closest neighbor, the cosmopolitan species *T. strigosum*. Growth of *T. strigosellum* sp. nov. at elevated temperatures (e.g. 33 °C) was significantly better than that of *T. strigosum*, which may imply a greater fitness in the tropical lowland forest ecosystems where the species occurs.

*Trichoderma* species have applications ranging from the production of enzymes and antibiotics (*H. jecorina/T. reesei*), to bioremediation of xenobiotic substances, and biological control of plant-pathogenic fungi and nematodes (Kubicek et al. 2011; Druzhinina et al. 2011). Previous studies on *Trichoderma* from neotropic regions focused on the isolation of strains with antifungal activity against pathogens of agro-industrially important crops, e.g. cacao (*Theobroma cacao*) and coffee (*Coffea* spp.) (Samuels et al. 2006a; Hanada et al. 2008; Mulaw et al. 2010). Our data confirm that the Amazon region harbors a rich pool of *Trichoderma* species, including yet undescribed species, which allows us to better understand their role in important ecological processes of these ecosystems such as nutrient cycling. Therefore, it is likely that further diversity explorations of this important group of fungi from these regions will yield significant data.

### *Taxonomy and description of* Trichoderma strigosellum *López*-*Q., W. Gams, BoekhoutandDruzhinina, sp. nov.*

#### Etymology

Lat. *strigosus* = meager, thin. *Strigosellus* = diminutive of *strigosus,* the epithet of the most closely related species.

Note: the Latin stem *strigosus* applied to *T. strigosellum* sp. nov. and *T. strigosum* may be used in two different interpretations, typifying the differences between the species and *striga* (Botanical Lat., a bristle-like hair) referring to the appearance of the conidiophore extensions in *T. strigosum*, and *strigosus* (Lat. meager, or boring of oratory) reflecting the plain appearance of the new species in lacking conidiophore extensions.

#### Mycobank

MB 804931.

### Holotype

In Herbarium Universidad de Antioquia as HUA 179963, with isotype in Herbarium CBS as CBS H-21054. Ex-type cultures CBS 102817 (=C.P.K. 3604), isolated from leaf litter exposed for 6 months in litter bags placed on forest floor in a *Pseudomonotes tropenbosii* (Dipterocarpaceaea) forest in Peña Roja, Department Amazonas, Colombia, July 1999. The isolate was collected by Carlos Lopez Quintero as CBS 102817.

### Morphology

**Fig. 6:**
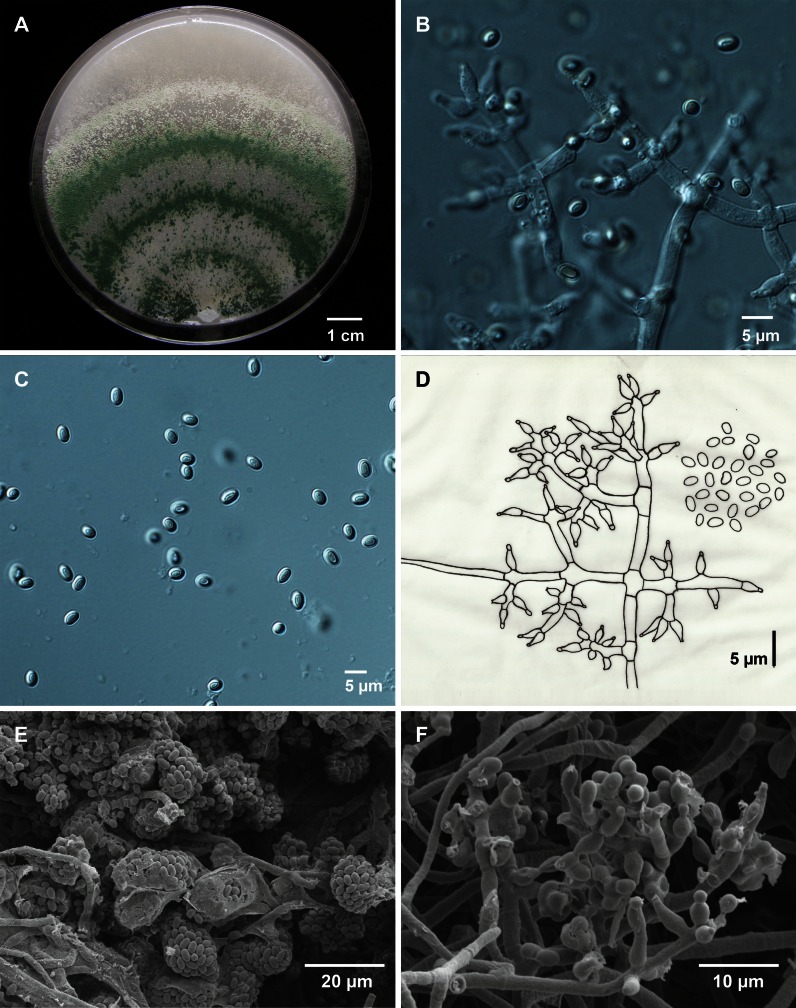
Morphology of *Trichoderma strigosellum* sp. nov. CBS 102817. **a**. Colony on cornmeal agar (CMA) at room temperature; **b**. Branching conidiophores and phialides on CMA; **c**. Conidia on CMA; **d**. Drawing of conidation and conidia from CMA; **e**. Low magnification of SEM image of spore clusters; **f**. SEM of hyphae, phialides and conidiogenesis

A new species is similar to *T. koningii* and *T. koningiopsis* but differentiated morphologically by much less developed aerial mycelium. Differing from its closest relative, *T. strigosum*, by complete absence of sterile conidiophore elongations and better growth at higher temperatures. *Colonies* on OA dark grey-green (Fig. 6) reaching 7–8 cm diameter after 5 days on CMA at 24 °C, and 9 cm after 6 days on CMD, SNA and OA at 27 °C, but only 0.1–0.4 cm on these three media at 36 °C. Submerged mycelium of young colonies irregularly and loosely branched, spreading radially; aerial mycelium with central conidiation after 4 days on CMD; zonate, with zones on OA approximately 15–18 mm distant, with scattered small pustules with deep green colour (27E8); growth on SNA sparse with loosely branched hyphae that form conidial heads, pustules hardly distinguishable, but after 7 days near the margin of the plate becoming distinct. Odor somewhat musty, but strain CBS 102805 had a coconut odor on OA.

Vegetative hyphae 2–8 μm wide, smooth- and thin-walled; but broader cells may have somewhat thickened cell walls, 20–60 × 6–8 μm (Fig. 6). *Conidiophores* profusely and pyramidally branched at right angles, main branches up to 2.5–5 μm wide, lacking sterile appendages (Fig. 6). Phialides flask-shaped, occurring in irregular clusters of 2–4, with swollen venter (2.5–3.5 μm wide) and a short neck, length 5–7(−10) μm (Fig. 6); terminal phialides more cylindrical and narrow than the intercalary ones, up to 11 μm long. *Conidia* ellipsoidal, smooth-walled, herbage-green in microscopic preparations; 3.5–4(–4.5) × (1.5–)2–2.5(–3.0) μm (Fig. 6). *Chlamydospores* globose, intercalary or terminal, hyaline, up to 8 μm diam. Optimum temperature for growth 24–27 °C, growth at 33 °C. Teleomorph unknown.

### Other material examined

CBS 102805, CBS 102806, CBS 102816, CBS 102818, all isolated from leaf litter exposed for 6 months in litter bags placed on forest floor in a *Pseudomonotes tropenbosii* (Dipterocarpaceae) forest in Peña Roja, Department Amazonas, July 1999; two isolates, López-Q. P1-2(25) and López-Q P4(166) were obtained from a secondary forest in Araracuara, Department Amazonas, Colombia. All isolates have been collected by Carlos Lopez Quintero.

### Comments

Among the species related to the *T. koningii* complex treated by Samuels et al. (2006b), *T. strigosellum* needs to be compared with other species having narrow conidia. In this respect, *T. strigosellum* resembles *T. koningii* and *T. koningiopsis,* but it does not form abundant aerial mycelium. Phylogenetically, *T. strigosellum* is a sister species to *T. strigosum*, but differs from the latter by the absence of sterile appendages and by smaller conidia and narrower phialides. Because *T. strigosum* has inconspicuous sterile or fertile conidiophore elongations, the species was placed by Bissett (1991) in section *Pachybasium.* The new species does not have such elongations. *T. strigosellum* can be reliably identified by high sequence similarity (>93 %) or identity of the *tef1* large intron sequence. Phylotypes of *tef1* large intron of *T. strigosum* share <90 % similarity with those of *T. strigosellum*. Note that no diagnostic coding regions were found for definitive species identification.

## Electronic supplementary material

Below is the link to the electronic supplementary material.

Supplementary material 1 (TIFF 13410 kb)

## References

[CR1] Atanasova L, Jaklitsch WM, Komon-Zelazowska M, Kubicek CP, Druzhinina IS (2010). Clonal species *Trichoderma**parareesei* sp. nov. likely resembles the ancestor of the cellulase producer *Hypocrea**jecorina*/*T.**reesei*. Appl Environ Microbiol.

[CR2] Atanasova L, Druzhinina IS, Jaklitsch WM (2013a) Two hundred *Trichoderma* species recognized based on molecular phylogeny. In: Mukherjee PK, Singh US, Horwitz BA, Schmoll M, Mukherjee M (eds.). *Trichoderma*: biology and applications. CABI, Nosworthy Way, Wallingford, Oxon, UK (in press)

[CR3] Atanasova L, Le Crom S, Gruber S, Coulpier F, Seidl-Seiboth V, Kubicek CP, Druzhinina IS (2013). Comparative transcriptomics reveals different strategies of *Trichoderma* mycoparasitism. BMC Genomics.

[CR4] Baker TR, Phillips OL, Malhi Y, Almeida S, Arroyo L, Di Fiore A, Erwin T, Higuchi N, Killeen TJ, Laurance SG, Laurance WF, Lewis SL, Monteagudo A, Neill DA, Vargas PN, Pitman NCA, Silva JNM, Martinez RV (2004). Increasing biomass in Amazonian forest plots. Philos Trans R Soc Lond B Biol Sci.

[CR5] Bissett J (1991). A revision of the genus *Trichoderma* III section pachybasium. Can J Bot.

[CR6] Chaverri P, Samuels GJ (2013). Evolution of habitat preference and nutrition mode in a cosmopolitan fungal genus with evidence of interkingdom host jumps and major shifts in ecology. Evolution.

[CR7] Chaverri P, Gazi R, Samuels GJ (2011). *Trichoderma amazonicum*, a new endophytic species on *Hevea brasiliensis* and *H. guianensis* from the Amazon basin. Mycologia.

[CR8] Coûteauxm M-M, Bottner P, Berg B (1995). Litter decomposition, climate and litter quality. TREE.

[CR9] De Souza JT, Pomella AWV, Bowers JH, Pirovani CP, Loguercio LL, Hebbar KP (2006). Genetic and biological diversity of *Trichoderma stromaticum*, a mycoparasite of the cacao witches’-broom pathogen. Phytopathology.

[CR10] Domsch KH, Gams W, Anderson T-H (2007). Compendium of soil fungi.

[CR11] Druzhinina IS, Kopchinskiy AG, Komon M, Bissett J, Szakacs G, Kubicek CP (2005). An oligonucleotide barcode for species identification in *Trichoderma* and *Hypocrea*. Fungal Genet Biol.

[CR12] Druzhinina IS, Kopchinskiy AG, Kubicek CP (2006). The first one hundred of *Trichoderma* species is characterised by molecular data. Mycoscience.

[CR13] Druzhinina IS, Komon-Zelazowska M, Kredics L, Hatvani L, Antal Z, Belayneh T, Kubicek CP (2008). Alternative reproductive strategies of *Hypocrea**orientalis* and genetically close but clonal *Trichoderma**longibrachiatum*, both capable of causing invasive mycoses of humans. Microbiology.

[CR14] Druzhinina IS, Kubicek CP, Komon-Zelazowska M, Mulaw TB, Bissett J (2010). The *Trichoderma**harzianum* demon: complex speciation history resulting in coexistence of hypothetical biological species, recent agamospecies and numerous relict lineages. BMC Evol Biol.

[CR15] Druzhinina IS, Komon-Zelazowska M, Atanasova L, Seidl V, Kubicek CP (2010). Evolution and ecophysiology of the industrial producer *Hypocrea**jecorina* (Anamorph *Trichoderma**reesei*) and a new sympatric agamospecies related to it. PLoS ONE.

[CR16] Druzhinina IS, Seidl-Seiboth V, Herrera-Estrella A, Horwitz BA, Kenerley CM, Monte E, Mukherjee PK, Zeilinger S, Grigoriev IV, Kubicek CP (2011). *Trichoderma*: the genomics of opportunistic success. Nat Rev Microbiol.

[CR17] Duivenvoorden JF, Lips JM (1993) Ecología del paisaje del Medio Caquetá. Memoria Explicativa de los Mapas (Landscape Ecology of the Middle Caquetá Basin; Explanatory Notes to the Maps). Tropenbos International, Wageningen, The Netherlands

[CR18] Duque AJ (2004) Plant diversity scaled by growth forms along spatial and environmental gradients. A study in the rain forest of NW Amazonia. PhD Thesis University of Amsterdam, The Netherlands. Tropenbos International, Wageningen, The Netherlands

[CR19] Friedl MA, Kubicek CP, Druzhinina IS (2008). Carbon source dependence and photostimulation of conidiation in *Hypocrea**atroviridis*. Appl Environ Microbiol.

[CR20] Gams W, Domsch KH (1967). Beiträge zur Anwendung der Bodenwaschtechnik für die Isolierung von Bodenpilzen. Arch Mikrobiol.

[CR21] Gams W, Verkleij GJM, Crous PW (2007). CBS Course of mycology.

[CR22] Hanada RE, Jorge Souza T, Pomella AWV, Hebbar KP, Pereira JO, Ismael A, Samuels GJ (2008). *Trichoderma martiale* sp. nov., a new endophyte from sapwood of *Theobroma cacao* with a potential for biological control. Mycol Res.

[CR23] Hättenschwiler S, Coq S, Barantal S, Handa IT (2011). Leaf traits and decomposition in tropical rainforests: revisiting some commonly held views and towards a new hypothesis. New Phytol.

[CR24] Hermosa MR, Grondona I, Iturriaga EA, Diaz-Minguez JM, Castro C, Monte E, García-Acha I (2000). Molecular characterization and identification of biocontrol isolates of *Trichoderma* spp. Appl Environ Microbiol.

[CR25] Hermosa MR, Keck EJ, Chamorro I, Rubio MB, Sanz L, Vizcaíno JA, Grondona I, Monte E (2004). Genetic diversity shown in *Trichoderma* biocontrol isolates. Mycol Res.

[CR26] Holdridge LR (1982). Ecología basada en zonas de vida.

[CR27] Holdridge LR, Grenke WC, Hatheway WH, Liang T, Tosi JA (1971). Forest environments in tropical life zones: a pilot study.

[CR28] Hoorn C, Wesselingh FP, ter Steege H, Bermudez MA, Mora A, Sevink J, Sanmartín I, Sanchez-Meseguer A, Anderson CL, Figueiredo JP, Jaramillo C, Riff D, Negri FR, Hooghiemstra H, Lundberg J, Stadler T, Särkinen T, Antonelli A (2010). Amazonia through time: andean uplift, climate change, landscape evolution, and biodiversity. Science.

[CR29] Hoyos-Carvajal L, Orduz S, Bissett J (2009). Genetic and metabolic diversity of *Trichoderma* from Colombia and adjacent neotropic regions. Fungal Genet Biol.

[CR30] Hoyos-Carvajal L, Orduz S, Bissett J (2009). Growth stimulation in beans (*Phaseolus vulgaris* L.) by *Trichoderma*. Biol Cont.

[CR31] Jaklitsch WM (2009). European species of *Hypocrea* part I the green-spored species. Stud Mycol.

[CR32] Jaklitsch WM (2011). European species of *Hypocrea* part II: species with hyaline ascospores. Fungal Divers.

[CR33] Jaklitsch WM, Samuels GJ, Dodd SL, Lu BS, Druzhinina IS (2006). *Hypocrea rufa/Trichoderma viride*: a reassessment, and description of five closely related species with and without warted conidia. Stud Mycol.

[CR34] Kopchinskiy A, Komon M, Kubicek CP, Druzhinina IS (2005). TrichoBLAST: a multilocus database for *Trichoderma* and *Hypocrea* identifications. Mycol Res.

[CR35] Kornerup A, Wanscher JH (1983). Methuen handbook of colour.

[CR36] Kraus GF, Druzhinina I, Gams W, Bissett J, Zafari D, Szakacs G, Koptchinski A, Prillinger HJ, Zare R, Kubicek CP (2004). *Trichoderma brevicompactum* sp. nov. Mycologia.

[CR37] Kreft H, Köster N, Küper W, Nieder J, Barthlott W (2004). Diversity and biogeography of vascular epiphytes in Western Amazonia, Yasuní, Ecuador. J Biogeogr.

[CR39] Kubicek CP, Herrera-Estrella A, Seidl-Seiboth V (2011). Comparative genome sequence analysis underscores mycoparasitism as the ancestral life style of *Trichoderma*. Genome Biol.

[CR40] Larkin MA, Blackshields G, Brown NP, Chenna R, McGettigan PA, McWilliam H, Valentin F, Wallace IM, Wilm A, Lopez R, Thompson JD, Gibson TJ, Higgins DG (2007). Clustal W and Clustal X version 2.0. Bioinformatics.

[CR41] Leaché AD, Reeder TW (2002). Molecular systematics of the eastern fence lizard (*Sceloporus undulatus*): a comparison of parsimony, likelihood and Bayesian approaches. Syst Biol.

[CR42] Lee CF, Hseu TH (2002). Genetic relatedness of *Trichoderma* sect. *Pachybasium* species based on molecular approach. Can J Microbiol.

[CR43] Londoño AC, Alvarez E, Forero E, Morton CM (1995). A new genus and species of *Dipterocarpaceae* from the Neotropics I introduction, taxonomy, ecology and distribution. Brittonia.

[CR44] López-Quintero CA, Straatsma G, Franco-Molano AE, Boekhout T (2012). Macrofungal diversity in Colombian Amazon forests varies with regions and with regimes of disturbance. Biodivers Conserv.

[CR45] Méndez MJ, Viteri SE (2007). Alternatives of biofertilization for sustainable onion bulb (*Allium cepa*) production in Cucaita, Boyacá. Agron Colomb.

[CR46] Migheli Q, Balmas V, Komon-Zelazowska M, Scherm B, Caria R, Kopchinskiy AG, Kubicek CP, Druzhinina IS (2009). Soils of a Mediterranean hotspot of biodiversity and endemism (Sardinia, Tyrrhenian Islands) are inhabited by pan-European and likely invasive species of *Hypocrea/Trichoderma*. Environ Microbiol.

[CR47] Mulaw TB, Kubicek CP, Druzhinina IS (2010). The rhizosphere of *Coffea**arabica* in its native highland forests of Ethiopia provides a niche for a distinguished diversity of *Trichoderma*. Fungal Divers.

[CR48] Nicholas KB, Nicholas HB Jr (1997) Genedoc: a tool for editing and annotating multiple sequence alignments. http://www.nrbsc.org/downloads/

[CR49] Nylander JAA, Wilgenbusch JC, Warren DL, Swofford DL (2008). AWTY (are we there yet?): a system for graphical exploration of MCMC convergence in Bayesian phylogenetics. Bioinformatics.

[CR50] Ortiz A, Orduz S (2000). In vitro evaluation of *Trichoderma* and *Gliocladium* antagonism against the symbiotic fungus of the leaf-cutting ant *Atta cephalotes*. Mycopathologia.

[CR51] Osono T (2005). Colonization and succession of fungi during decomposition of *Swida controversa* leaf litter. Mycologia.

[CR52] Osono T, Takeda H (2002). Comparison of litter decomposing ability among diverse fungi in a cool temperate deciduous forest in Japan. Mycologia.

[CR53] Phillips OL, Baker TR, Arroyo L (2004). Pattern and process in Amazon tree turnover, 1976–2001. Philos Trans R Soc Lond B.

[CR54] Pitman NAC, Terborg J, Silman MR, Nuñez P, Neill DA, Cerón CE, Palacios WA, Aulestia M (2001). Dominance and distribution of tree species in upper Amazonian Terra Firme forests. Ecology.

[CR55] Posada D (2003) Using MODELTEST and PAUP* to select a model of nucleotide substitution. Current Protocols in Bioinformatics John Wiley & Sons, Inc. doi: 10.1002/0471250953.bi0605s00 10.1002/0471250953.bi0605s00 18428705

[CR56] Powers JS, Montgomery RA, Adair EC, Brearly FQ, DeWalt SJ, Castanho CT, Chave J, Deinert E, Ganzhorn JU, Gilbert ME, González-Iturbe JA, Bunyavejchewin S, Grau HR, Harms KE, Hiremath A, Iriarte-Vivar S, Manzane E, de Oliveira AA, Poorter L, Rnmanamanjato J-B, Salk C, Varela A, Weiblen GD, Lerdau MT (2009). Decomposition in tropical forests: a pan-tropical study of the effects of litter type, litter replacement and mesofaunal exclusion across a precipitation gradient. J Ecol.

[CR57] Ronquist F, Huelsenbeck JP (2003). MRBAYES 3: bayesian phylogenetic inference under mixed models. Bioinformatics.

[CR58] Rozas J, Sánchez-DelBarrio JC, Messeguer X, Rozas R (2003). DnaSP, DNA polymorphism analyses by the coalescent and other methods. Bioinformatics.

[CR59] Samuels GJ (2006). *Trichoderma*: systematics, the sexual state, and ecology. Phytopathology.

[CR60] Samuels GJ, Dodd SL, Lu B-S, Petrini O, Schroers H-J, Druzhinina IS (2006). The *Trichoderma**koningii* aggregate species. Stud Mycol.

[CR61] Samuels GJ, Suarez C, Solis K, Holmes KA, Thomas SE, Ismaiel A, Evans HC (2006). *Trichoderma theobromicola* and *T. paucisporum*: two new species isolated from cacao in South America. Mycol Res.

[CR62] Swofford DL (2002) PAUP*: Phylogenetic Analysis Using Parsimony (*and Other Methods), Version 4.0b10. Sinauer Associates, Sunderland, MA

[CR63] Talavera G, Castresana J (2007). Improvement of phylogenies after removing divergent and ambiguously aligned blocks from protein sequence alignments. Syst Biol.

[CR65] Taylor JW, Jacobson DJ, Kroken S, Kasuga T, Geiser DM, Hibbett DS, Fisher MC (2000). Phylogenetic species recognition and species concepts in fungi. Fungal Genet Biol.

[CR66] Teertstra WR, van der Velden GJ, de Jong JF, Kruijtzer JA, Liskamp RM, Kroon-Batenburg LM, Müller WH, Gebbink MF, Wösten HA (2009). The filament-specific Rep1-1 repellent of the phytopathogen *Ustilago maydis* forms functional surface-active amyloid-like fibrils. J Biol Chem.

[CR67] Ter Steege H, Pitman N, Sabatier D, Castellanos H, Van Der Hout P, Daly DC, Silveira M, Phillips O, Vasquez R, Van Andel T, Duivenvoorden J, de Oliveira AA, Ek R, Lilwah R, Thomas R, Van Essen J, Baider C, Maas P, Mori S, Terborgh J, Nuñez Vargas P, Mogollón H, Morawetz W (2003). A spatial model of tree α-diversity and tree density for the Amazon. Biodivers Conserv.

[CR68] Thompson JD, Gibson TJ, Plewniak F, Jeanmougin F, Higgins DG (1997). The Clustal X windows interface: flexible strategies for multiple sequence alignment aided by quality analysis tools. Nucleic Acids Res.

[CR69] Tobón Marín C (1999) Monitoring and modeling hydrological fluxes in support of nutrient cycling studies in Amazonian rain forest ecosystems. PhD Dissertation, University of Amsterdam, Amsterdam, The Netherlands. Tropenbos International, Wageningen, The Netherlands

[CR70] Tuomisto H, Ruokolainen K, Yli-Halla M (2003). Dispersal, environment, and floristic variation of western Amazonian forests. Science.

[CR71] Veerkamp J, Gams W (1983). Los hongos de Colombia VIII. Some new species of soil fungi from Colombia. Caldasia.

[CR72] White TJ, Bruns T, Lee S, Taylor J, Innis MA, Gelfand DH, Sninsky JJ, White TJ (1990). Amplification and direct sequencing of fungal ribosomal RNA genes for phylogenetics. PCR Protocols: a guide to methods and applications.

